# Microfabrication of Thermoplastic Polypropylene Surface Structures via Thermal Imprinting for Controlling the Adhesion of Easy Peel Package

**DOI:** 10.1021/acsomega.3c04671

**Published:** 2023-09-14

**Authors:** Charinee Winotapun, Thidarat Makmoon, Chuanchom Aumnate, Dumrong Thanomjitr, Wuttipong Rungseesantivanon, Hiroshi Ito

**Affiliations:** † National Metal and Materials Technology Center, National Science and Technology Development Agency, Thailand Science Park, Pathum Thani 12120, Thailand; ‡ Metallurgy and Materials Science Research Institute, Chulalongkorn University, Bangkok 10330, Thailand; § Center of Excellence in Responsive Wearable Materials, Chulalongkorn University, Bangkok 10330, Thailand; ∥ Faculty of Engineering, Graduate School of Organic Materials Science, Yamagata University, Yamagata 990-0021, Japan

## Abstract

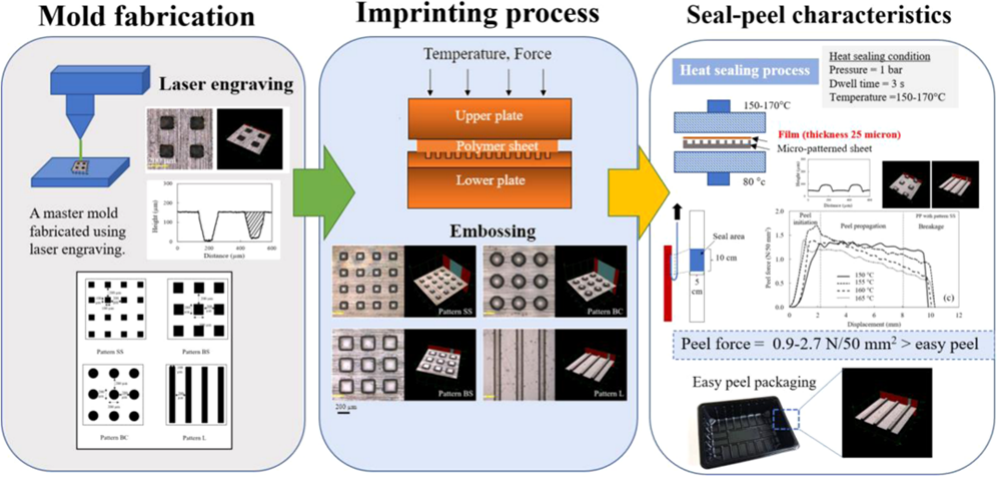

Micropatterns were fabricated on polypropylene (PP) surfaces using the hot embossing technique with various temperatures ranging from 160 to 175 °C and applying force conditions from 100 to 300 N. To evaluate the replication quality, an effective filling ratio of 1 indicates that the volume of the formed pattern is similar to the mold cavity volume. From the results, the filling ratio increased with increasing the embossing temperature. For instance, under a constant force of 100 N, the filling ratio of polypropylene (PP) with small square arrays (pattern SS) increased from 0.08 to 0.41 when the embossing temperature was raised from 160 to 175 °C, respectively. With the increase of applied force, the filling ratio also increased. At an imprinting temperature of 175 °C and an applied force of 300 N, the highest effective filling ratio that was achieved was approximately 0.99. Furthermore, the effect of PP with different melt flow indexes (MFIs) on the filling ratio was investigated. For food packaging applications, a micropatterned PP sheet was heat-sealed with a biaxially oriented polypropylene (BOPP) film. The micropatterned PP sheet demonstrated easy-opening properties by varying sealing contact areas and micropattern geometries between the sheet and the BOPP film. All micropatterned PP sheets with an MFI of 25 g/10 min exhibited an easy peel property with adhesive failure characteristics at a heat-sealing temperature of 150 °C and a dwell time of 3 s. There was no residue on the PP substrate surface. The overall findings are beneficial in understanding the hot embossing technology for fabricating micropatterns on polymer surfaces, and it can be applied in an easy peel property for packaging applications.

## 1 Introduction

The fabrication of micropatterns or microstructures on substrate surfaces is an important issue in many applications such as microlens arrays for optical devices,^1^ microneedles,^2−4^ and self-cleaning surfaces for biomedical devices.^5−10^ The microstructure is replicated from a rigid mold onto a polymer substrate. Many proposed techniques include thermal imprinting processes (hot embossing^11−14^ and roll-to-roll hot embossing^15^), injection molding,^16−20^ and nanoimprint lithography.^21,22^ Micro hot embossing is a novel low-cost technique for microstructure replication. Advantages of hot embossing technology are high precision, high replication quality on a micro-/nanoscale, simple operation, and low cost.^11−13^ In this technique,^23^ a polymer substrate is positioned on the upper or lower plate, and a micropatterned master mold is attached on the opposite side. In principle, hot embossing of amorphous polymers involves heating the polymer substrate above its glass-transition temperature (*T*_g_). A microstructured master mold is then pressed on the polymer substrate, followed by transfer of the pattern. After a sufficient contact time, the system is cooled to lower than *T*_g_ before separating the master mold from the substrate (demolding). Semicrystalline polymers are heated to nearly the melting temperature (*T*_m_) at a viscous flow state followed by applying force and demolding. The imprinting quality of the hot embossing method is affected by various processing parameters including temperature, force, and time.^23,24^ Besides, the melt flow index (MFI) of a polymer is one of the important parameters affecting replication quality. The MFI corresponds to the polymer’s average molecular weight (*M*_w_), which can improve production quality. Understanding the polymer flow behavior is vital information for achieving a high replication quality.

Polypropylene (PP) has superior mechanical properties, with high heat resistance and low price, making it a popular material for packaging applications.^25,26^ The primary objective of packaging is to protect the product from external physical, chemical, and biological impacts. Packages are usually prepared by heat sealing between a plastic container and a film. In a heat-sealing system, the contact area between the two material surfaces is heated by a heat-sealing machine under sufficient time and pressure.^27−29^ Adhesion of the two materials is promoted due to the polymer-chain entanglement of molecular segments diffusing across the interface of the two surfaces. A bond can be rapidly formed by melting the plastic and then resolidifying.^30^ Temperature, pressure, and contact time are the key parameters that influence heat-sealing strength.^31^ There are two typical heat seal characteristics, including weld seal and peelable seal. Weld seal develops high seal strength for the packaging as resistance against leakages. Peelable packaging is usually formed by coating with a heat-sealing material or multilayer film fabrication. By designing incompatibility between two materials in the sealant layer, weak bonding at the interface is achieved.^32−34^ Peel force ranging from 0.9 to 2.7 N/50 mm^2^ is considered a peelable seal (or easy-open).^34^ The total heat-sealed contact area was 50 mm^2^, which is calculated from the sample width of 5 mm and a seal flat bar width of 10 mm.

Easy peel packaging is now more convenient for consumers and is in high demand in a variety of end-use industries such as electronics, pharmaceuticals, food, and beverage. Due to changes in consumer behavior and lifestyle, they prefer ready-to-eat, frozen, and processed foods. As a result, supermarket retail packages of fresh-cut fruit, ready-to-eat food, and meat products have increased. The development of packaging with an easy peel property has become more challenging for packaging manufacturers who want to balance a consumer-friendly easy peel property with an effective high seal strength.

Liewchirakorn et al.^34^ developed poly(lactic acid) (PLA) blended with a poly(butylene adipate-*co*-terephthalate) (PBAT) film with peelable properties. The peelable film heat-sealed with a PLA tray was produced by incorporating 20% PBAT into the PLA film. Sängerlaub et al.^35^ studied polyethylene (PE) blended with various compositions of polybutene-1 (PB-1) as a sealant layer film. They stated that the incompatibility between polyolefin and PB-1 led to low intermolecular bonding, resulting in an easy peel property.

Copious research has focused on the development of a multilayer peelable lidding film.^35−38^ Nevertheless, multilayer films are difficult to recycle because each film layer may contain a wide range of materials and polymers with variable properties. As a result, developing recyclable monomaterial plastic films and plastic trays is a challenge. Scant attention has been paid to peelable plastic trays or containers by creating micropatterns on the surface of the plastic tray lid heat-sealed with the same material as the plastic film. Interfacial science and engineering have been extensively researched in order to achieve various functionalities based on micro-/nanosurface structures. The structure and underlying mechanisms of the adhesive substrates have attracted the interest of scientists for decades.^39,40^ Few studies have investigated heat-sealed contact areas and contact geometries between polymer substrates and films with basic mechanisms and easy peel properties for packaging. Therefore, this research investigated the effects of hot embossing temperature, applied force, and the type of materials on the replication quality. Square arrays and circular arrays were fabricated on the PP surface. Rheological factors related to the molecular weight of materials were also evaluated, and a framework for determining polymer viscosity, complex shear modulus, frequency sweep, and temperature sweep measurements of polypropylene is presented. Discussions emphasize how molecular weight, imprinting temperature, and polymer viscosity affect the filling ratio during the hot embossing process. The fabrication of micropatterns on a plastic PP sheet was systematically studied for packaging applications. Micropatterned PP sheets were heat-sealed with commercial biaxially oriented poly(propylene) (BOPP) films at various sealing temperatures. Effects of adhesion contact areas and micropattern geometries on the peelable properties are presented and discussed.

## 2 Materials and Methods

### 2.1 Materials

Commercial-grade homopolymer polypropylene resins with melt flow indexes (MFIs) of 8 and 25 g/10 min (230 °C and 2.16 kg) were purchased from HMC Polymer Company Limited, Thailand, while the commercial biaxially oriented polypropylene (BOPP, homopolymer polypropylene) film of 25 μm thickness was supplied by A.J. Plast Public Company Limited, Thailand.

### 2.2 Differential Scanning Calorimetry (DSC)

Polymer crystallinity was analyzed with a differential scanning calorimeter (DSC1, Mettler Toledo AG, Canada). Samples of 5–8 mg were examined under nitrogen gas flow at a heating rate of 10 °C/min. The crystallinity percentage of the samples was investigated by using the endothermic melting peaks. The enthalpy of melting for 100% crystallinity of PP was 209 J/g.^41^ At least three samples were tested for the crystallinity percentage calculation with mean and standard deviations recorded.

### 2.3 Rheological Measurements

Dynamic rheological testing was performed using a strain-controlled rotational rheometer (ARES G2, TA Instrument), equipped with 25 mm diameter parallel plates and a 1 mm gap for all measurements. Temperature sweep testing was performed at a frequency of 1 rad/s at 160 and 200 °C, with the measurement investigated at a fixed strain of 0.1% over a frequency range of 0.06–600 rad/s. Storage modulus (*Ǵ*), loss modulus (*G* ˝), and complex viscosity (η*) were measured.

### 2.4 Cast Film Extrusion

The PP sheet was fabricated by cast sheet extrusion (Haake PolyLab OS RheoDrive7, Thermo Scientific) through a T-die with a screw speed of 60 rpm and a die gap of 0.3 mm. Optimal barrel (zones 1–4), adapter (zone 5), and die set temperatures (zones 6–7) were 60, 180, 190, 200, 210, 230, and 230 °C, respectively, with the chilled roll set at 40 °C. The parameters remained constant for all fabrications. The output rate was 3 kg/h, and the thickness of the PP sheet was 300 μm.

### 2.5 Mold Fabrication

**Figure 1:**
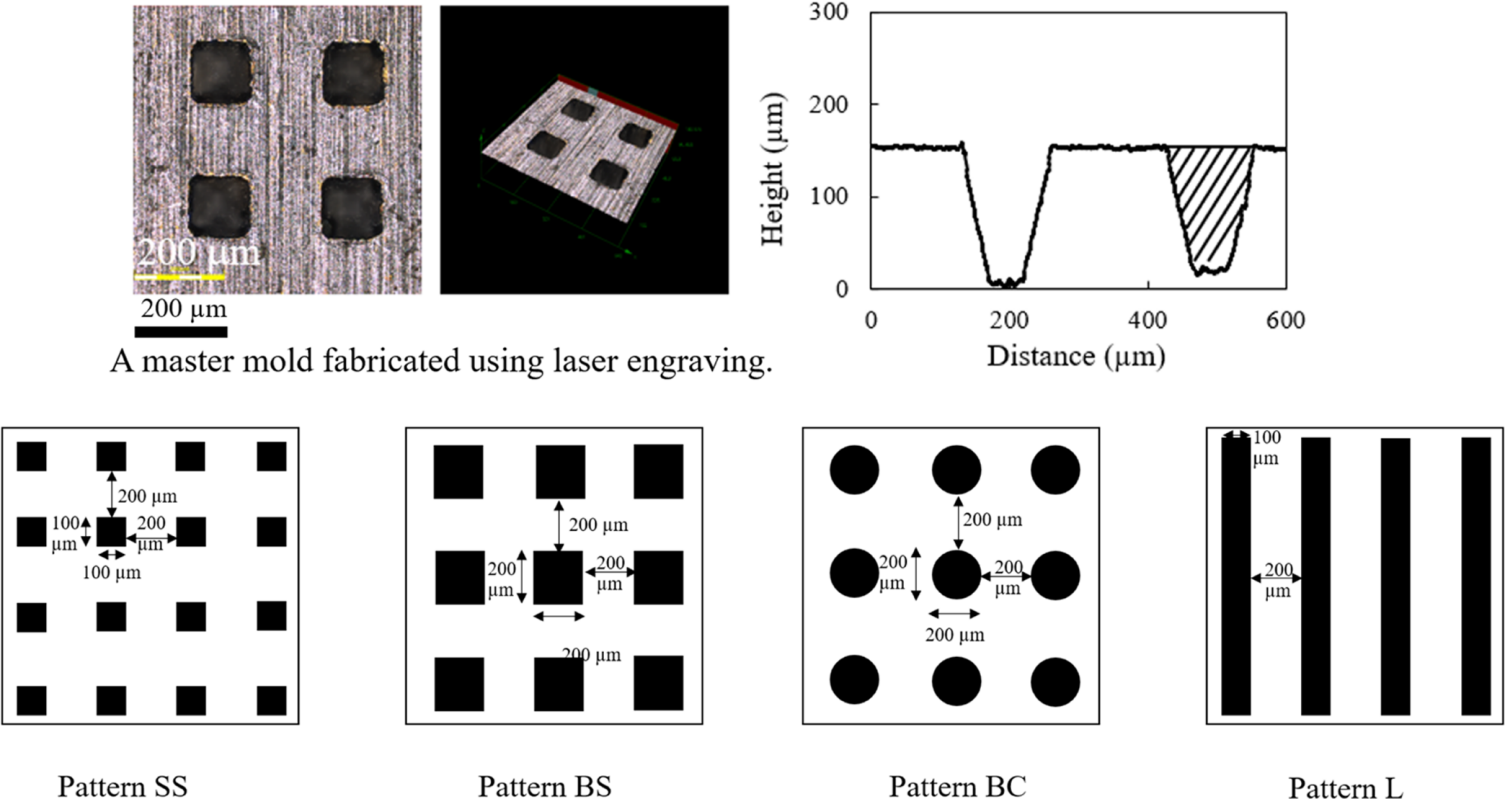
Microstructure patterns on the master mold with different micropatterns including square arrays with square block size 100 μm, height 50 μm, and pitch distance 200 μm (pattern SS), square arrays with square block size 200 μm, height 50 μm, and pitch distance 200 μm (pattern BS), circular arrays with circular diameter block size 200 μm, height 50 μm, and pitch distance 200 μm (pattern BC), and line arrays with line width 100 μm, height 50 μm, and pitch distance 200 μm (pattern L) fabricated using laser engraving.

The master metal mold was ablated using a nanosecond laser with a wavelength and pulse duration of 1064 nm and 4 ns, respectively, and a focal spot diameter of 25 μm. Geometries and surface profiles of pattern cavities are illustrated in Figure 1.

### 2.6 Imprinting Process

Micropattern arrays on the master mold were imprinted onto the PP surface at temperatures from 160 to 175 °C via a hot embossing machine. The PP sheet was preheated for 3 min, followed by an applied force of 100–300 N for 1 min. After 1 min, the force was released, and the samples were removed to cool to 30 °C for 3 min, followed by separating the master mold from the PP substrate.

### 2.7 3D Laser Scanning Confocal Microscope

The formed micropattern surface profiles were analyzed using a laser scanning confocal microscope (LEXT OLS4100, Olympus, Japan). Micropattern volume on the PP sheet was determined. At least five samples were evaluated.

### 2.8 Heat Seal Testing

The micropatterned PP substrate was heat-sealed with commercial BOPP films using a heat-sealing machine (Lako Tool SL2, Lako Tool and Manufacturing, Inc.) with an upper and lower heat-sealing flat seal bar. The upper plate temperature was varied from 145 to 165 °C, while the lower plate temperature was fixed at 80 °C. Sealing pressure and dwell time were 1 bar and 3 s, respectively. The samples were subsequently cooled to room temperature.

### 2.9 Peeling Force Measurement

All peel tests were performed on a universal testing machine (model 5943, Instron), according to ASTM F88-00. The peel force was measured at 180°, following the I-peel test method. Gauge lengths and widths of the samples were 25 and 5 mm, respectively. The seal flat bar width was 10 mm. Therefore, the total sealing contact area was 50 mm^2^, which corresponds to a film sample width of 5 mm and a flat bar width of 10 mm. Samples were tested at a crosshead speed of 200 mm/min using a 100 N load cell. Peel force–elongation curves were recorded for five specimens. The average peel force for each specimen was determined between 25 and 80% of elongation in the peel force curve (or plateau-like region). Five specimens per set were tested and averaged.

### 2.10 Statistical Analysis

One-way analysis of variance (ANOVA) (Minitab software for Windows, version 21) was used to investigate the statistical data. Results were compared by post hoc Tukey tests with significant difference at *p* < 0.05 (95% confidence interval) and stated as mean ± standard deviation.

## 3 Results and Discussion

### 3.1 Thermal Properties of PP Sheets

**Figure 2:**
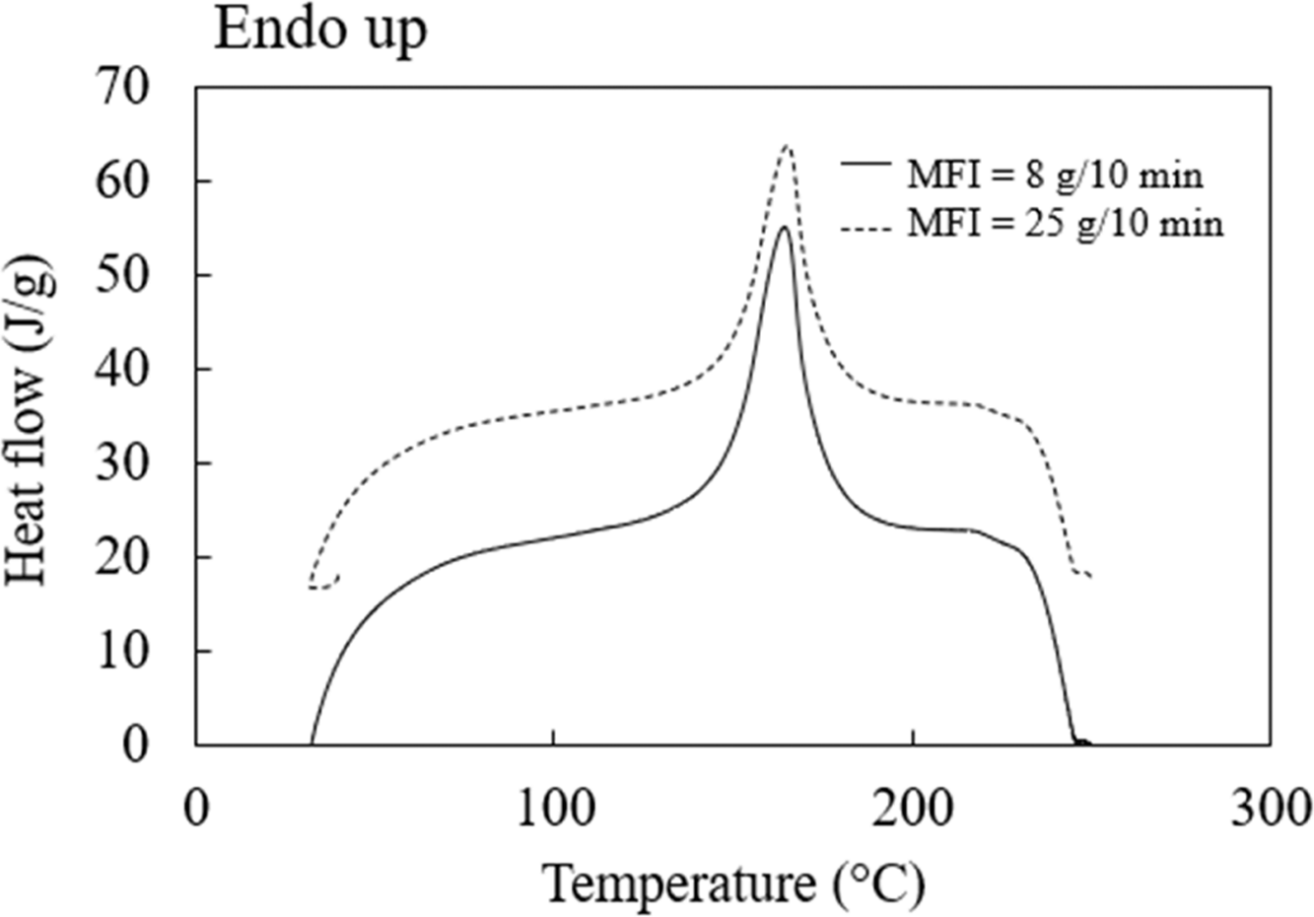
DSC thermograms of PP with MFI values of 8 and 25 g/10 min.

DSC thermograms of PP sheets with melt flow indexes (MFIs) of 8 and 25 g/10 min are presented in Figure 2. The endothermic melting peaks of both PP sheets were similar, with onset, peak, and endset melting temperatures of 153, 165, and 172 °C, respectively. The crystallinity of PP sheets with MFIs of 8 and 25 g/10 min were 33.19 and 32.08%, respectively, as can be seen in Table 1.

**Table 1:** Thermal Properties of PP with MFIs of 8 and 25 g/10 min

MFI	*T*_m_ (°C)	crystallinity (%)
8	165	33.19
25	165	32.08

### 3.2 Effect of Applied Force and Embossing Temperature

**Figure 3:**
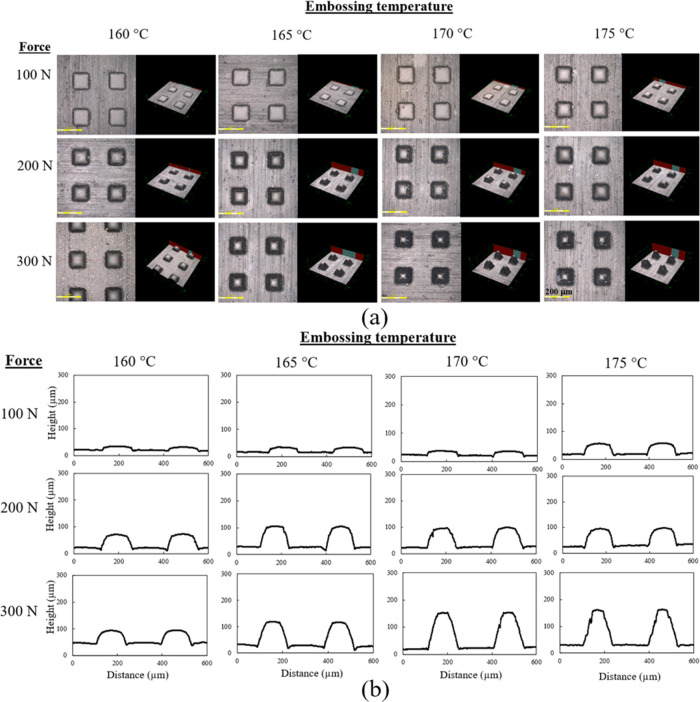
(a) Three-dimensional (3D) laser confocal microscope photographs and (b) surface profiles of micropattern arrays fabricated at embossing temperatures of 160–175 °C under three applied forces: 100, 200, and 300 N.

PP sheets with MFI values of 8 and 25 g/10 min were embossed at various temperatures and applied forces, and surface profiles and volumes of micropattern arrays were investigated. Microstructure patterns on the master molds were used to study the effect of the imprinting temperature and applied force. For the pattern SS, the volume of one cavity of the master mold was 1.7 × 10^6^ μm^3^. In the hot embossing process, the temperature normally ranges between the glass-transition temperature (*T*_g_) and the melting temperature (*T*_m_) of the polymer.^42^ Consequently, a temperature close to the melting point of PP was used to increase the fluidity and fill the micropatterned mold. Replication qualities and surface profiles of a micropatterned PP with an MFI of 25 g/10 min using various embossing conditions are shown in Figure 3a,b. Increasing the embossing temperature and applied force showed a greater micropattern on the PP surface. The volume of the protruding microstructure pattern was estimated. The effective filling ratio was calculated as the formed pattern volume divided by the mold cavity volume, as presented in eq 1. In an ideal state, the formed pattern volume should be equal to the volume of the mold cavity with an effective filling ratio of 1.
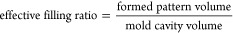
1Changes in the applied force on the replication quality of the micropattern showed results similar to changes in the embossing temperature. The effective filling ratio of micropatterned PP with an MFI of 25 g/10 min increased with increasing the embossing temperature, as shown in Table 2. At a constant embossing temperature of 160 °C, the effective filling ratio increased significantly from 0.08 to 0.68 on increasing the applied force from 100 to 300 N. Using a higher force promoted the flow of PP to fill the master mold. High applied force also increased the efficiency of thermal transfer, with an improved surface contact area between the PP sheet and the master mold.^43^

**Table 2:** Effective Filling Ratio of Micropattern Arrays with the Pattern SS on PP with MFIs of 8 and 25 g/10 min Fabricated Using Embossing Temperatures of 160 to 175 °C under Three Applied Forces of 100, 200, and 300 N^a^

	PP with MFI of 8 g/10 min			PP with MFI of 25 g/10 min		
embossing temperature (°C)	100 N	200 N	300 N	100 N	200 N	300 N
160	0.08 ± 0.0^cC^	0.21 ± 0.03^cC^	0.40 ± 0.15^bB^	0.08 ± 0.02^cC^	0.44 ± 0.02^cB^	0.68 ± 0.02^bA^
165	0.16 ± 0.01^bcD^	0.31 ± 0.01^cC^	0.53 ± 0.01^bB^	0.16 ± 0.03^cD^	0.66 ± 0.05^bA^	0.69 ± 0.03^bA^
170	0.26 ± 0.02^bC^	0.53 ± 0.01^bB^	0.83 ± 0.03^aA^	0.29 ± 0.07^bC^	0.57 ± 0.07^bB^	0.87 ± 0.11^aA^
175	0.41 ± 0.09^aC^	0.80 ± 0.08^aB^	0.99 ± 0.04^aA^	0.47 ± 0.03^aC^	0.79 ± 0.02^aB^	0.99 ± 0.02^aA^

a Data are reported as mean ± standard deviation. Lowercase superscripts (a–c) show significant differences (*p* < 0.05) along columns, while uppercase superscripts (A–C) show significant parameter differences (*p* < 0.05) in each row.

To study the effect of imprinting temperature, a constant applied force was applied. As shown in Figure 3, the effective filling ratios of the micropattern arrays were close to 1 at a force and an embossing temperature of 300 N and 175 °C, respectively. The effective filling ratio of the micropattern increased with temperature. At a constant force of 300 N, the effective filling ratio increased significantly from 0.68 to 0.99 when the embossing temperature was raised from 160 to 175 °C due to a reduction in the viscosity of PP as the mold temperature increased, allowing the polymer to more easily fill the microstructured master mold and form the micropattern arrays.^43^ Thus, the embossing temperature and applied force are both important factors for replicating the micropatterned polymer.

### 3.3 Effect of PP with Different Melt Flow Indexes Based on Rheological Properties

Factors influencing micropattern replication include the temperature and pressure used, as well as the polymer melt flow index (MFI). The MFI is a crucial parameter that determines the resistance to the flow of a polymer melt at an applied temperature under pressure for a period of time through a fixed orifice. The measurement procedure specifies precise orifice dimensions, fixed temperature, and load mass (pressure) as the mass of polymer flowing through the orifice in 10 min. Several previous articles used an empirical model to describe the relationship among MFI, flow curves, and average molecular weight (*M*_w_) of polyethylene (PE) and polypropylene (PP) thermoplastics.^44−46^ The Hagen–Poiseuille equation is often used to explain the flow of liquid passing through a fixed-size orifice to correlate the relationship between MFI and *M*_w_(45) as follows:
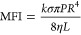
2where σ is the polymer density (g/cm^3^), *k* is 600 (s/10 min), and *P*, *R*, η, and *L* are pressure (g/cm·s^2^), radius of the die orifice (cm), melt viscosity (g/cm·s), and orifice length (cm), respectively. Thus, the melt viscosity of the polymer is directly correlated with the MFI. Assuming a continuous flow for extremely low shear ranges, the relationship between zero-shear viscosity (η_0_) and *M*_w_ can be estimated using the power law of Mark–Houwink as follows:
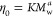
3where *K* and *a* are constants depending on the type of polymer. For PP, the *a* value is around 3.4.^45^ The MFI is inversely proportional to the melt viscosity for linear polymers with similar polydispersities^1^ and is related to *M*_w_ as shown in eq 4.
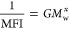
4where *G* and *x* are constants depending on the polymer type. Bremner et al.^45^ determined the *x* value as between 3.4 and 3.7. Therefore, the inverse of the MFI can be related to the average polymer molecular weight using the power function (*M*_w_^3.4^).^44,45^ However, when polymers have variable branches and polydispersity indices, this correlation becomes tenuous.

The polymer melt flow index was measured according to ASTM D1238 or ISO1133, utilizing the temperature of each polymer’s completely melted state. The recommended temperature for PP is 230 °C with a load of 2.16 kg. However, the appropriate temperature should be observed for the hot embossing process. For example, two PP samples with MFI values of 8 and 25 g/10 min were embossed using the pattern SS. At temperatures lower than 175 °C, the replication quality of micropattern arrays improved with increasing MFI, as shown in Table 2. At an embossing temperature of 160 °C and an applied force of 300 N, the effective filling ratio increased from 0.40 to 0.68 when the MFI increased from 8 to 25 g/10 min, respectively, and was explained by the flowability of PP at various temperatures. Higher MFI values of PP improved the replication quality of the micropattern arrays due to the lower viscosity, allowing the melted polymer to easily enter the mold cavity.

After cooling, solidification is associated with crystallization and subsequent volume shrinkage. Several studies^47,48^ identified crystallization as an important factor causing shrinkage and leading to dimension inaccuracy. Crystallization causes shrinkage, as the molecular chains rearrange into a structure. A higher degree of PP crystallinity with an MFI of 8 g/10 min (33.19%) (Table 1) led to higher shrinkage and contributed to differences in the filling ratio between MFI values of 8 and 25 g/10 min.

However, the melt flow index and viscosity did not impact the filling capacity when the temperature was high enough to melt the polymer, leading to a filling ratio close to 1. The hot embossing process could not be quantified at temperatures greater than 175 °C (data not shown) because the viscosity of the PP was too low. The protruding microstructures on the PP surface improved with an increasing melt flow index when the temperature was below 175 °C. PP with a high MFI had a high effective filling ratio of micropattern arrays due to the low viscosity that flowed easily.^49,50^ The MFI represents polymer melt flow properties at a specific temperature and pressure.^51,52^

To illustrate the changes in flow behavior driven by polymer viscosity in the hot embossing method, PP was imprinted at two MFI values. Replication of PP with different MFI values can be explained based on rheological measurements. The viscosity of a polymer is a crucial parameter. Therefore, a full thermomechanical characterization of the two polymers was performed using oscillatory shear testing with a rotational rheometer. To determine changes in viscosity, the temperature was varied from 160 to 200 °C, with a melting temperature of PP of 165 °C.

Modulus and complex viscosity values of a polymer depend on the molecular weight (*M*_w_), with high *M*_w_ polymers showing a high modulus and complex viscosity. High *M*_w_ polymers improve the mechanical properties of finished products, but low *M*_w_ polymers offer easier processing. Therefore, *M*_w_ is an important factor for the embossing ability.

**Figure 4:**
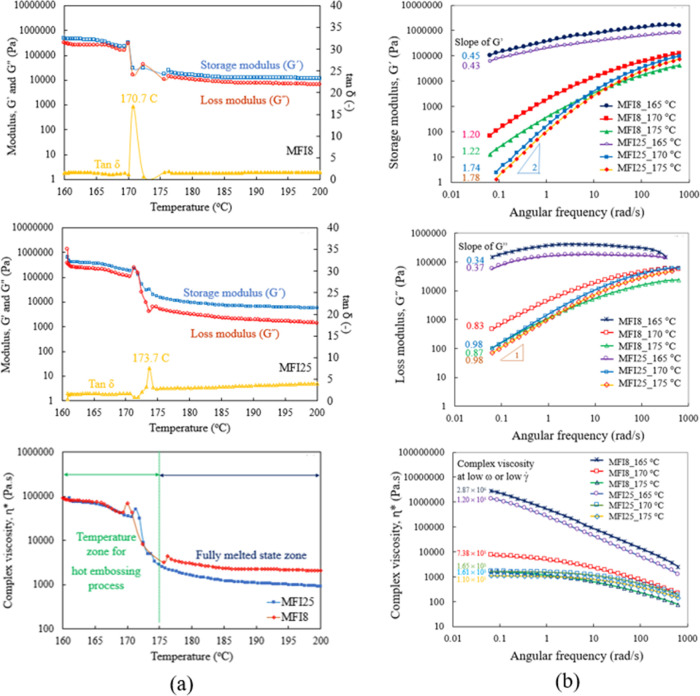
Storage and loss modulus values as a function of temperature (a) PP with an MFI of 8 g/10 min, PP with an MFI of 25 g/10 min, and complex viscosity as a function of temperature and (b) storage modulus, loss modulus, and complex viscosity as a function of frequency of the polypropylene sheet with MFIs of 8 and 25 g/10 min at temperatures of 165, 170, and 175 °C.

Thermo-rheological curves of storage modulus (*Ǵ*), loss modulus (*G*), and loss tangent (tan δ = *G*″/*Ǵ*) as a function of temperature covering 160–200 °C for PP with MFIs of 8 and 25 g/10 min are shown in Figure 4a. A change from a solid-like behavior, *Ǵ* > *G*″, to a liquid-like behavior, *G*″ > *Ǵ*, was observed with an increase in temperature. The temperature at which both moduli cross, *Ǵ* = *G*″ or peak of tan δ, is called the transition temperature. This was measured at 170.7 and 173.7 °C for PP with MFIs of 8 and 25 g/10 min, respectively. Results in Figures 2 and 4a–c show that below the melting temperature of PP, both storage and loss modulus values were maximum. Upon heating, both storage and loss modulus values decreased since less force was required for deformation. The storage modulus gradually decreased when the temperature increased from 160 to 165 °C, followed by a sharp decrease with an increase in temperature. The transition temperature between 170 and 173 °C for the two PP samples with different MFI values corresponded to the melting temperature obtained by the static temperature scan from DSC, as shown in Figure 2. This transition reflected the order–disorder temperature of microphase-separated polymers, as the temperature at which the ordered structure disintegrates and a homogeneous polymer melt forms.^53^

Storage and modulus characteristics can be explained by the mobility of the polymer molecular segments. At lower temperatures, polymer molecules oscillate less because of the low kinetic energy. As the temperature increases, the kinetic energy of the molecules increases, resulting in an increased mobility of the molecular segments. This enlarges the free volume between the molecular segments and lowers the storage modulus.^54,55^Figure 4a demonstrates the complex viscosity of PP with two different MFI values as a function of temperature. The complex viscosity of polymers gradually decreased from 160 to 165 °C due to increasing kinetic energy. When the kinetic energy was high enough to drive segmental motions, the modulus and complex viscosity of PP rapidly decreased at 165–175 °C with a significant increase of segmental motion at the melting temperature. Thus, the temperature for the hot embossing process should range between 160 and 175 °C to maintain soft characteristics with a suitable filling property.

To compare PP with MFIs of 8 and 25 g/10 min, sweep tests were carried out at a fixed strain of 0.1% over a frequency range of 0.06–600 rad/s. Figure 4b illustrates the storage modulus, loss modulus, and complex viscosity as a function of frequency measured at embossing temperatures of 165, 170, and 175 °C. Mathematical models are used to describe and quantify the rheological, dynamical, and structural properties of a sample. The Maxwell model is the most basic and straightforward. Normally, at sufficiently low frequencies in the so-called terminal zone of a double logarithmic plot, *G*′ and *G*″ increase linearly with slopes of 2 and 1, respectively.^56^ The viscoelastic response of the polymer at a low angular frequency was strongly dependent on phase morphology. At low frequencies, the modulus of PP with an MFI of 8 g/10 min at 175 °C and that of PP with an MFI of 25 g/10 min at 170–175 °C both exhibited a typical terminal behavior observed in homopolymer melts characterized by *G*″ ∼ ω and *Ǵ* ∼ ω^2^, ^57^ where ω is the angular frequency. However, when the temperature decreased to 165 °C, the slopes of *Ǵ* and G̋ for both PP samples with MFIs of 8 and 25 g/10 min deviated from 2 and 1, respectively, as shown in Figure 4b. Between 160 and 165 °C, the behavior of incomplete melt parts of the polymer molecules can occur at a low frequency in the terminal zone. As a result, slopes of both *Ǵ* and G̋ decreased when the temperature was reduced from 175 to 165 °C. These results correspond to the thermo-rheological curves illustrated in Figure 4a. The fully melt state began at 170–175 °C. The equation for relaxation or equilibration time (τ_e_) can be represented as
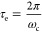
5where ω_c_ is the crossover frequency of *Ǵ* and *G̋* at the high-frequency limit.^58^ Thus, relaxation time is related to a predetermined contact period before separation of the master mold and the substrate (demolding). Relaxation times of PP with MFIs of 8 and 25 g/10 min reached 7.20 and 13.96 s, respectively (Table 3), with an incomplete melt at 165 °C that caused underfilling of the mold cavity. When the temperature increased to 170 °C, the relaxation time of these two PP samples decreased to 0.14 s for PP with an MFI of 8 g/10 min and to 0.04 s for PP with an MFI of 25 g/10 min. When the polymer was in the molten state at 175 °C, the relaxation time was low enough to completely fill the mold cavity, with relaxation values of PP with MFIs of 8 and 25 g/10 min at 0.72 and 0.03 s, respectively.

**Table 3:** Rheological Factors of PP with MFIs of 8 and 25 g/10 min at Temperatures of 165–175 °C, as Derived from Figure 4b and Equation 5

	PP with MFI of 8 g/10 min		PP with MFI of 25 g/10 min	
embossing temperature (°C)	complex viscosity at low angular frequency, η^a^ (Pa·s)	relaxation time, τ_e_ (s)	complex viscosity at low angular frequency, η^a^ (Pa·s)	relaxation time, τ_e_ (s)
165	2.87 × 10^6^	7.20	1.20 × 10^5^	13.96
170	7.38 × 10^3^	0.14	1.61 × 10^3^	0.04
175	1.65 × 10^3^	0.72	1.11 × 10^3^	0.03

a Standard deviation less than 5% from three replicated data.

The complex viscosity value, especially at a low angular frequency of PP with MFIs of 8 and 25 g/10 min, decreased with temperature increment (Figure 4b). Moreover, for PP with an MFI of 8 g/10 min, the complex viscosity value at 175 °C was significantly lower than the complex viscosity value at 170 °C due to incomplete replication (effective filling ratio of 0.83) at an embossing temperature of 170 °C and force of 300 N. There was an insignificant difference in the complex viscosity between both PP grades at 175 °C. Complete replications of both PP sheets using an embossing temperature of 175 °C and force of 300 N were achieved, with a protruded volume of PP replication with melt flow indices of 8 and 25 g/10 min at similar levels. These results identified the critical parameters affecting the high replication quality for manufacturing.

**Figure 5:**
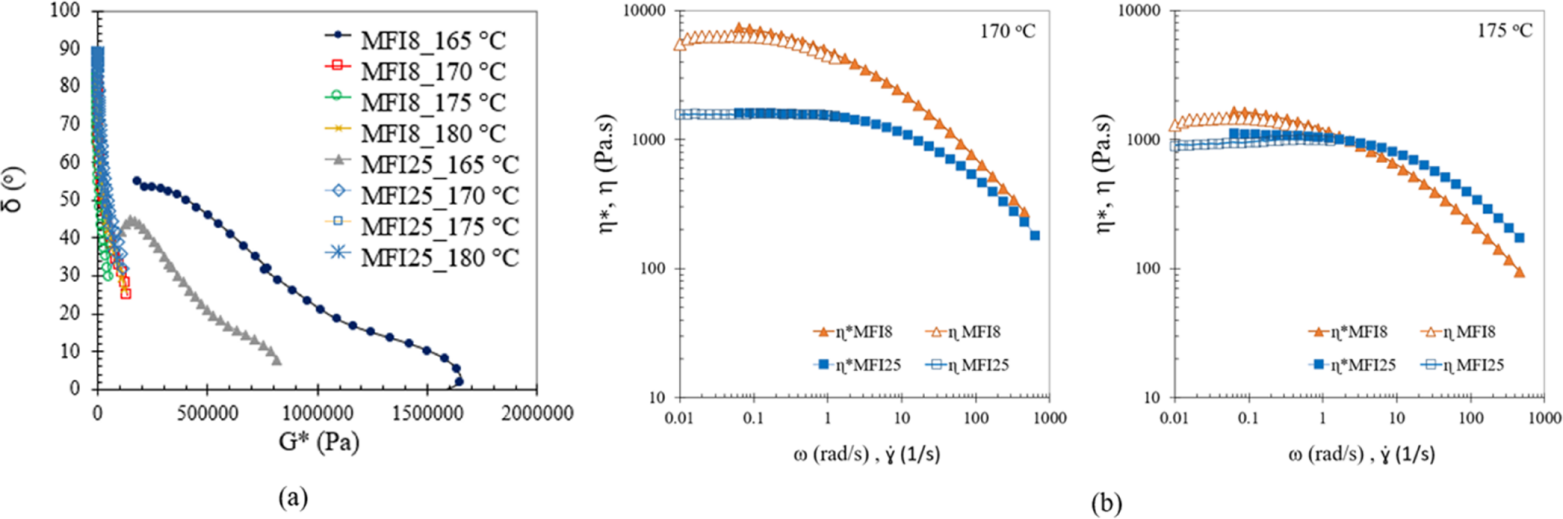
(a) Phase angle (δ) as a function of complex modulus (G*) of a polypropylene sheet with MFIs of 8 and 25 g/10 min at temperatures of 165, 170, 175, and 180 °C and (b) frequency (ω) dependence of complex viscosity (η*) and shear rate (γ̇) dependence of steady shear viscosity (η) for a polypropylene sheet with MFIs of 8 and 25 g/10 min at temperatures of 170 and 175 °C.

Rheological data in Figure 5a show the phase angle (δ) as a function of the complex modulus (G*) of the polypropylene sheet with MFIs of 8 and 25 g/10 min at temperatures of 165, 170, 175, and 180 °C. Reduction in mobility (δ minimum) at 165 °C was observed. Unmelted crystals that inflicted further barriers to motion are the most likely reason for the delay in the true terminal zone. At 165 °C, PP with different MFI values behaved as a multiphase polymer system with thermo-rheological complexity. At this temperature, the thermomobility of PP with an MFI of 8 g/10 min was higher than PP with an MFI of 25 g/10 min. Therefore, unmelted parts of PP with an MFI of 25 g/10 min were lower than PP with an MFI of 8 g/10 min, corresponding to the higher viscosity of PP with an MFI of 8 g/10 min, as shown in Figure 4b. However, thermo-rheological simplicity increased with temperature. The slope of δ did not change significantly from 170 to 180 °C for both grades of PP, suggesting that at 165 °C, the phase angle of PP with MFIs of 8 and 25 g/10 min strongly deviated from the molten state compared to 170–180 °C.

The Cox and Merz rule^59^ states that shear rate dependence of steady shear viscosity η (γ̇) equals the frequency dependence of complex viscosity η* (ω). As presented in Figure 5b, at 170 and 175 °C, the relation between complex viscosity η* (ω) and shear rate dependence viscosity η (γ̇) of PP with MFIs of 8 and 25 g/10 min follows the Cox and Merz rule. Therefore, complex viscosity η* (ω), shown in Figure 4b, was used to estimate the zero-shear viscosity η_0_ (γ̇), corresponding to eqs 3 and 4.

At 175 °C, PP with an MFI of 8 g/10 min and, at 170–175 °C, PP with an MFI of 25 g/10 min showed no significant change in complex viscosity at low ω or zero-shear viscosity. As a result, no significant difference in the filling ratio approaching 1 using these temperatures and applying a force of 300 N was found in the hot embossing process.

### 3.4 Seal-Peel Characteristics

**Figure 6:**
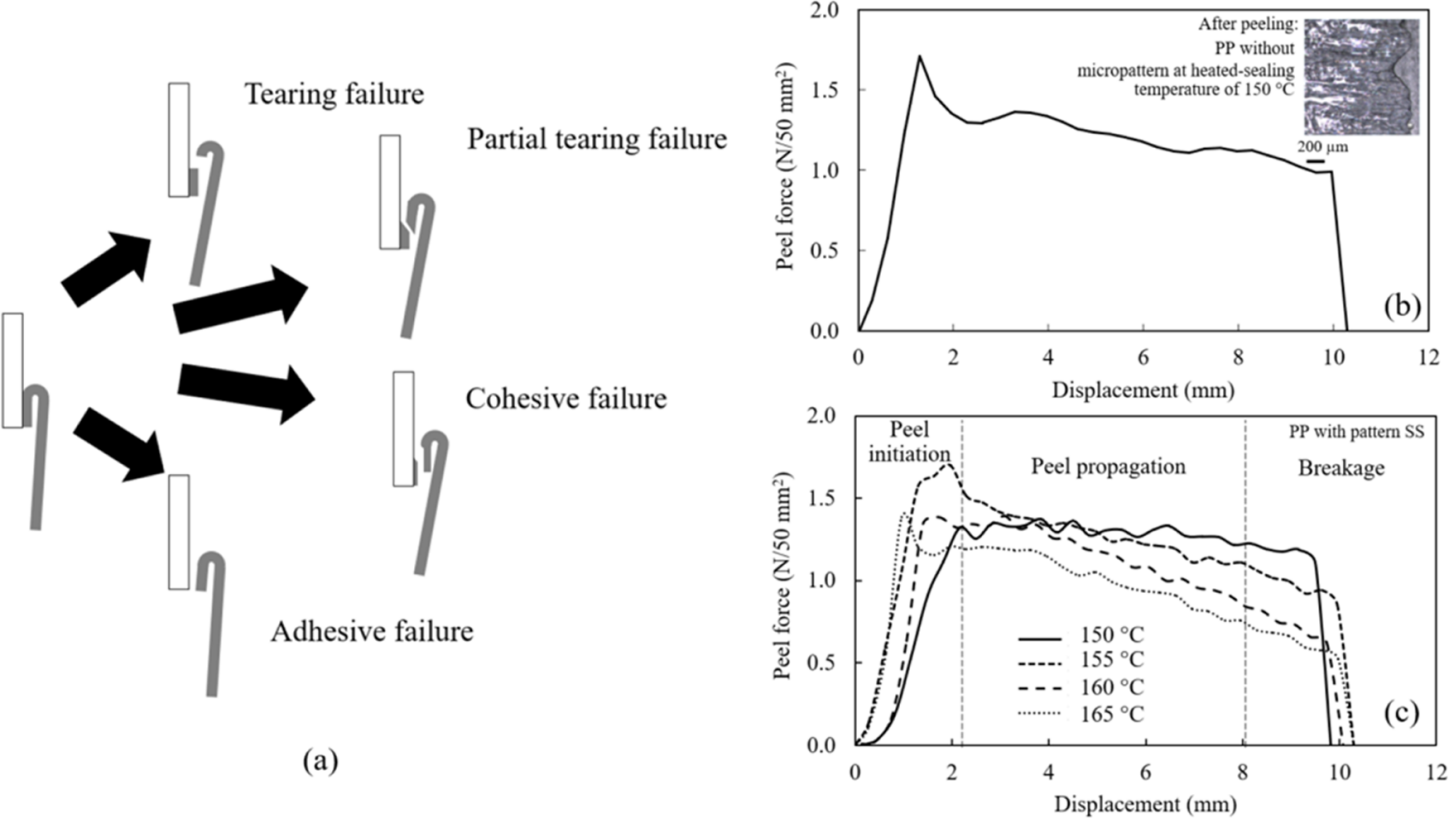
(a) Four typical peeling failure characteristics including tearing, partial tearing, cohesive, and adhesive failure. (b) Peeling curve of PP without and (c) with the pattern SS at various heat-sealing temperatures (pressure of 1 bar and dwell time of 3 s).

To demonstrate monomaterial packaging application, micropatterned PP sheets were sealed with a commercial biaxially orientated polypropylene (BOPP) film using several temperatures. The effects of the contact area and geometry were investigated. In the heat-sealing technique, the polymer interface was melted, and the molecular chains diffuse and form entanglements under heating and pressing between two heat seal bars. After removing the heat seal bar, the sealed sheet/film cools, solidifies, and crystallizes.^27^ Peel force is the ratio of force and contact area required to separate the polymer substrate and the film. The peeling test was performed at room temperature. As can be seen in Figure 6a, four typical peeling failure characteristics are tearing, partial tearing, cohesive, and adhesive failure. Tearing occurs when the film breaks during the peeling process because of high heat-sealing strength between the two materials. Partial tearing failure results from a peel followed by tearing of the film. In cohesive failure, the film peels off from the substrate during the test, with peel force normally lower than for both tearing and partial tearing failures. There are some residues remaining on both peeled surfaces of the substrate and films. Adhesive failure occurs at the interface between the film and the substrate, leading to the lowest peel strength results. No residue remains on both peeled surfaces of the substrate and films.^27^ In addition, peeling characteristics rely on the sealing conditions. The PP sheet without and with a micropattern cannot be sealed with the PP film below the heat-sealing temperature of 150 °C (at a constant pressure of 1 bar and a dwell time of 3 s). At a heat-sealing temperature of 150 °C, the PP sheet with no micropattern heat-sealed with the BOPP film showed tearing characteristics, whereas all micropatterned PP sheets revealed an easy peel property. The large adhesion area between the interface of the PP sheet without the micropattern and the BOPP film caused the tearing characteristics. There was no peeling during the peeling process due to a strong seal strength (or the seal strength was greater than the inherent tensile strength of the film), as can be seen from the peeling curve of the PP sheet in Figure 6b. These results concurred with those of Mazzola et al.,^60^ who reported that when the contact area increased, molecular chain entanglements between the PP sheet and the BOPP film exhibited a strong joint at the interfacial zone.

To study the effect of sealing conditions, for example, a PP sheet with the pattern SS and an MFI of 25 g/10 min was used to investigate heat-sealing conditions. The PP sheet was heat-sealed with a BOPP film from 150 to 165 °C at a constant pressure and a dwell time of 1 bar and 3 s, respectively. The peeling curve of the PP sheet without and with the micropattern heat-sealed with the BOPP film using various temperatures is shown in Figure 6c. Average peel forces were between 25 and 80% of elongation (in the range of peel propagation or plateau-like region). Peel force in the range of 0.9–2.7 N/50 mm^2^ is considered a peelable seal (easy-open).^34^ The total heat-sealed contact area was 50 mm^2^, which is calculated from the sample width of 5 mm and the seal flat bar width of 10 mm.

**Figure 7:**
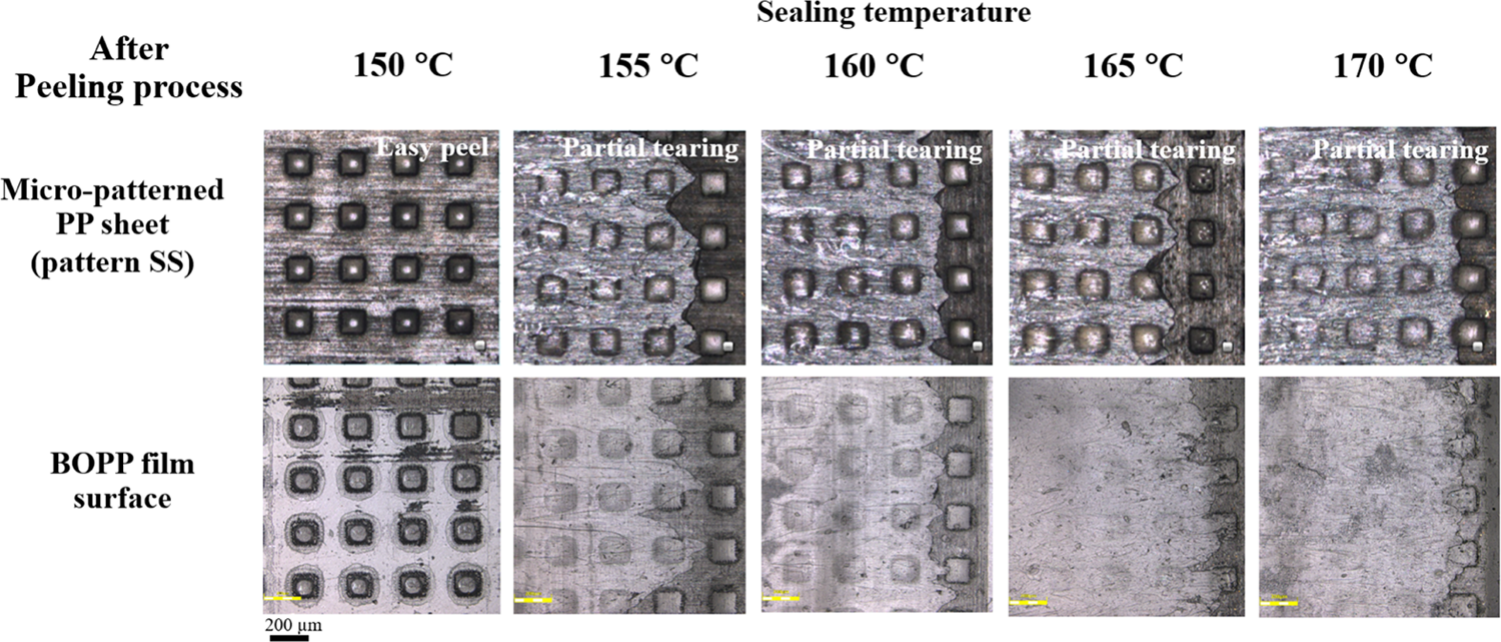
Optical microscope images of the micropatterned PP sheet (MFI of 25 g/10 min) with the pattern SS and BOPP film after the peeling process.

At a heat-sealing temperature of 150 °C, the peel force of the PP sheet with the pattern SS was 1.3 ± 0.2 N/50 mm^2^, which is in the range of peelable seal. The micropatterned PP sheet showed easy peel properties with adhesive failure characteristics. No residue remains on the substrate surface, as can be seen in Figure 7. Above the sealing temperature of 150 °C, a combination of peel and tear failure was evident as a partial tearing failure mechanism. The lidding film started with a peel, followed by the tearing of the peel arm. BOPP film residues were observed on micropatterned PP. Under an increasing temperature, the applied pressure enhanced the molecular contact of the molten film surfaces. With sufficient time, polymer-chain segments diffused across the interface and created molecular entanglements between polymer molecules in the interfacial zone.^61^ After cooling, recrystallization occurred. Hence, high temperatures resulted in high seal strength.^61−66^

Comparing the MFI of PP with a similar pattern SS as square arrays (square block size 100 μm, height 50 μm, and pitch distance 200 μm), peeling characteristics of PP with MFIs of 8 and 25 g/10 min were studied. The PP sheet with an MFI of 25 g/10 min heat-sealed with the BOPP film at 150 °C showed an easy peel characteristic. However, low melt flow showed the failure characteristic of partial tearing under all sealing conditions. High adhesion and a stronger seal were observed in PP with an MFI of 8 g/10 min. The melt flow index is related to molecular weight (*M*_w_); a polymer with a low MFI corresponds to a high molecular weight (*M*_w_). In the heat-sealing process, high-molecular-weight chains typically show higher interfacial adhesion than low-molecular-weight chains because long chain diffusion encourages entanglements across the interface between the two materials at high temperature and sufficient dwell time.^64^ This result was consistent with that of Ilhan et al.^66^ They stated that the polymer sample with a low melt flow index had a high seal strength because of the low MFI, which was attributed to the high average molecular weight and the presence of long molecules in the same types of polymers. Longer molecules can produce more entanglement. After sealing, they will recover their original shape at a certain level and form new entanglements at the seal interface. Hence, materials with a lower MFI have higher seal strengths.

**Figure 8:**
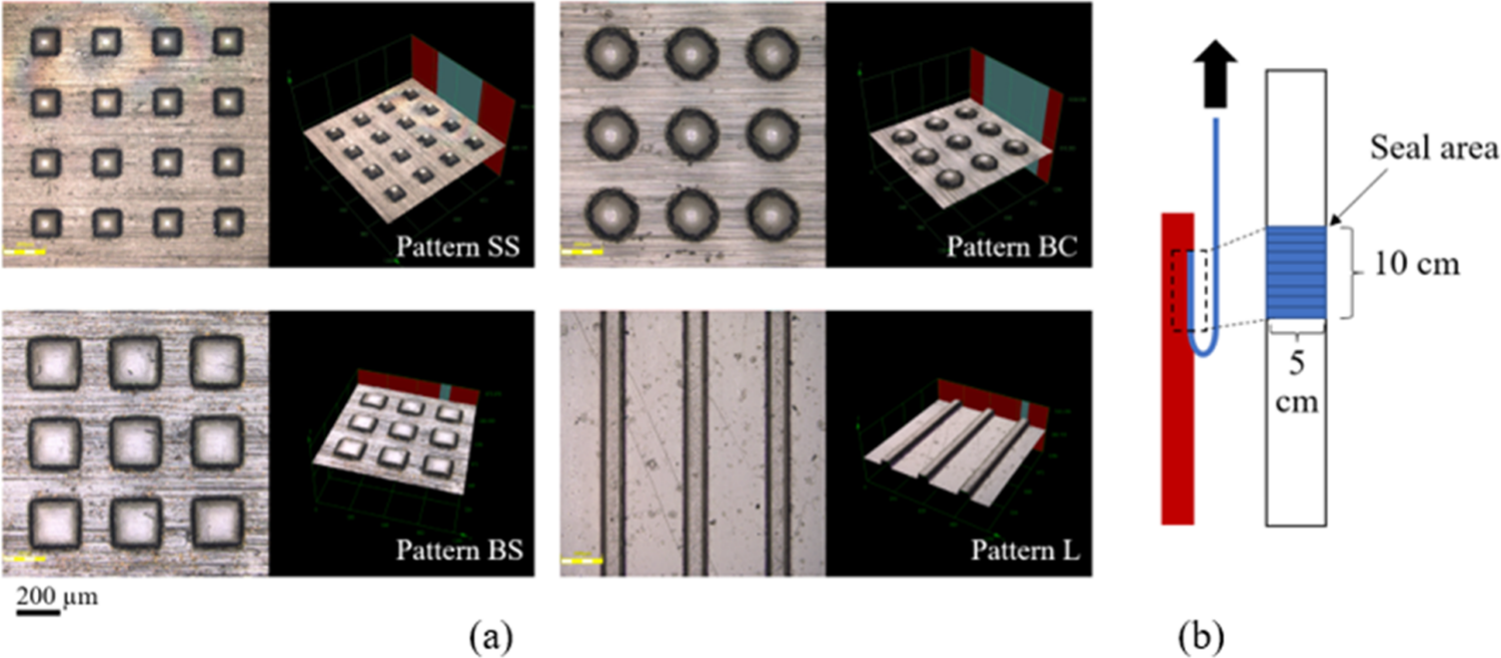
(a) Microstructure of different micropatterns (SS, BS, BC, and L) and (b) schematic view of the peeling process.

**Figure 9:**
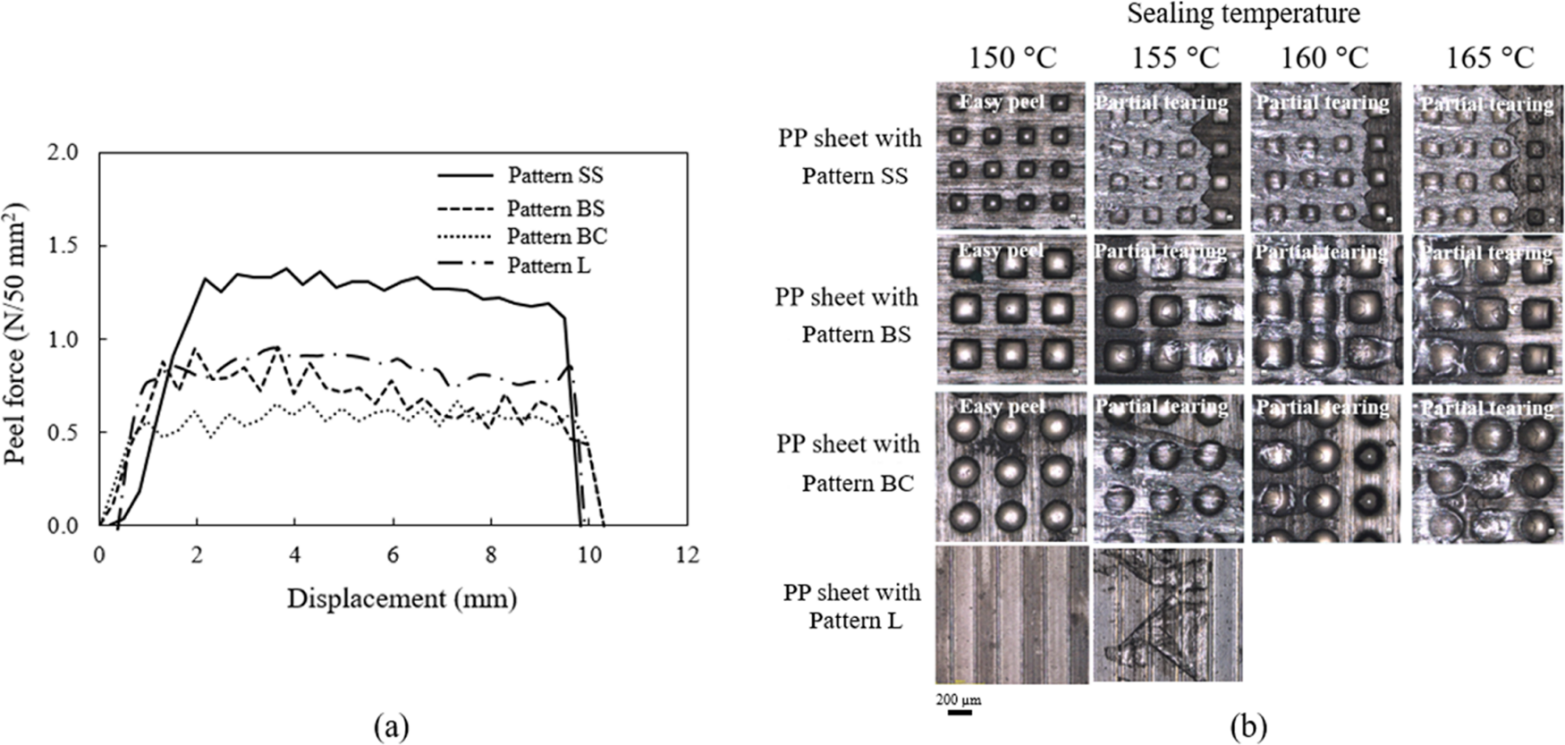
(a) Peeling curve of the micropatterned PP sheet heat-sealed with the BOPP film using 150 °C and (b) the optical microscope images of the micropatterned PP sheet (MFI of 25 g/10 min) with various micropatterns after the peeling process.

As for the effect of contact areas and geometries on sealing properties, Table 4 shows micropattern contact areas and geometries of patterns SS, BS, BC, and L used in this study. The microstructure of different micropatterns (SS, BS, BC, and L) and the schematic view of the peeling process are shown in Figure 8a,b. To evaluate the effect of the adhesion contact area, PP sheets with a similar contact geometry of square arrays (patterns SS and BS) were investigated. At a sealing temperature of 150 °C, peel forces of PP with patterns SS and BS were 1.3 ± 0.2 and 0.9 ± 0.1 N/50 mm^2^, respectively. Peel force of the PP sheet with the pattern SS was higher than that of the PP sheets with the pattern BS, as illustrated in Figure 9a. For the total seal test area of 50 mm^2^ (sample width of 5 mm and flat bar width of 10 mm, seen in Figure 8b), the contact area between the film and PP with the pattern SS (adhesion area 6.6 × 10^6^ μm^2^ (∼13% of the total contact area) and 661 micropillars) was smaller than for pattern BS (adhesion area 14.9 × 10^6^ μm^2^ (∼30% of the total contact area) and 372 micropillars), but micropatterns on PP with the pattern SS showed a higher number of micropillars than the pattern BS. Surprisingly, the number of pillars played an important role in the adhesive force, leading to a high peel force of PP sheets with the pattern SS. Adhesion was improved by splitting the contact area into many smaller ones. As a partial tearing failure mechanism, a combination of peel and tear failure was evident above the sealing temperature of 150 °C. Film residues were observed on the surface of the micropatterned PP, as depicted in Figure 9b.

**Table 4:** Shapes and Characteristics of Micropatterns on the PP Sheet with Patterns SS, BS, BC, and L^a^

pattern description	shape parameters	MFI	projected contact area (μm^2^)/contact elements	number of contact elements/seal area (50 mm^2^)	projected contact area (μm^2^)/seal area (50 mm^2^)	spacing surface area (μm^2^) /seal area (50 mm^2^)	peel force (N/50 mm^2^)
nonpatterned		25				50.0 × 10^6^	
SS	square arrays (square block size 100 μm, height 50 μm, and pitch distance 200 μm)	8	31,400	661	6.6 × 10^6^	43.4 × 10^6^	partial tearing
		25					1.3 ± 0.2^a^
BS	square arrays (square block size 200 μm, height 50 μm, and pitch distance 200 μm)	25	40,000	372	14.9 × 10^6^	35.1 × 10^6^	0.9 ± 0.1^b^
BC	circular arrays (circular diameter block size 200 μm, height 50 μm, and pitch distance 20 μm)	25	31,400	372	11.7 × 10^6^	38.3 × 10^6^	0.6 ± 0.0^c^
L	line pattern arrays (line pattern width 100 μm, height 50 μm, and pitch distance 200 μm)	25	500,000	27	13.5 × 10^6^	36.4 × 10^6^	0.9 ± 0.2^b^

a Sealing temperature of 150 °C, pressure of 1 bar, and dwell time of 3 s. Data are reported as mean ± standard deviation. Different superscripts within the same row indicate statistically significantly different values (*p* < 0.05).

PP sheets with patterns BS and BC were selected to demonstrate different contact geometries. The contact area and the number of micropillars on PP sheets with the pattern BS (square arrays) and the pattern BC (circular arrays) were almost the same (total micropillars of 372 pillars in a total sealing area of 50 μm^2^). The peel force relies on the geometry of the contact pillar. The peel force of the PP sheet with the pattern BS (square arrays) was higher than that of the PP sheet with the pattern BC (circular arrays). For the square arrays, separation began at the rim of the square and then expanded toward the center, while the film sealed with circular arrays was found to be more easily separated, with the peel force lower than for square arrays.

Generally, in the experiments, the initial curve in the peel test was sharp. Therefore, the peel test focuses mainly on the peel strength (*F*_(L)_) at each position along the width (*w*). The failure energy (*S_t_*) can be expressed by
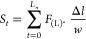
6where Δ*l* is an arbitrary unit of distance for each calculation (m) and *L*_*n*_ is the peel width (m). Therefore, from this equation, the peel strength depends on the specimen width. When the film was peeled, separation between the film and the sheet first started at the smallest width at the corner of the circular contact point, resulting in low peel force in the pattern BC, while separation between the film and the sheet with square arrays began at the rim width of the square contact point, leading to a high peel force.

From the previous experiment, it can be concluded that controlling the contact surface area and the contact geometry of the micropattern can be designed to provide the easy peel function of packaging. However, in the beginning, a micropattern was a pillar. For use as packaging, it can be seen that there are no hermetic seals for the food products, and atmospheric oxygen and carbon dioxide are able to penetrate the container. As a result, it may be unsuitable for use in food packaging, considering the permeability of oxygen and carbon dioxide. As a result, the study of a long square line (pattern L) shape on the PP substrate was developed that can be sealed without gaps at the joints. The PP sheet with a long square line (pattern L) was heat-sealed with the BOPP film at a heat-sealing temperature of 150 °C at a pressure and dwell time of 1 bar and 3 s, respectively. The micropatterned PP sheet with the pattern L (total long square lines of 27 lines and adhesion area of 13.5 × 10^6^ μm^2^, approximately 27% of the total sealing area of 50 μm^2^) showed easy peel properties with adhesive failure characteristics. The peel force of 0.9 ± 0.2 N/50 mm^2^ is considered peelable (easy-open). Even though the PP sheet with long square lines only has 27 total long block lines, a high adhesion area was observed. Because the total contact area of patterns L and BS was nearly identical, the peel force for both patterns was approximately 0.9 N/50 mm^2^. Comparing patterns L and SS, the pattern SS showed a higher peel force than the pattern L. The seal strength was improved by splitting of the contact area into many smaller ones. The overall findings are beneficial in understanding the critical parameters of hot embossing technology for fabricating micropatterns on polymer surfaces. The results can be applied in an easy peel property for packaging applications.

## 4 Conclusions

Micropattern design on the polypropylene (PP) sheet surface was successfully developed as an easy peel property for packaging applications. Micropatterns were fabricated on PP surfaces using the hot embossing technique at temperatures between 160 and 175 °C under an applied force ranging from 100 to 300 N. The filling ratio increased with embossing temperature and applied force. In addition, rheological factors strongly depended on temperature. Thermo-rheological behavior is an effective tool to define the appropriate temperature for hot embossing. By controlling the heat-sealing contact area between the micropatterned PP sheet and the BOPP film, the microstructure morphology of the PP sheet showed a reduction of peel strength compared to the neat PP sheet without micropatterns at sealing temperature 150 °C and pressure 1 bar for 3 s. Our findings contribute to a better understanding of the fabrication of micropatterns on polymer surfaces by using hot embossing technology and the application of micropatterns on polymer substrates with easy peeling properties for packaging.
